# The Effects of Terrestrial and Aquatic Activities on Foot Health: A Comparative Analysis of Podiatric Disorders

**DOI:** 10.3390/healthcare13070695

**Published:** 2025-03-21

**Authors:** Ana María Pérez Pico, Julia Villar Rodríguez, Joao Belo, María Victoria Cáceres-Madrid, Marina Fontán-Jiménez, Raquel Mayordomo

**Affiliations:** 1 Department of Nursing, University Centre of Plasencia, University of Extremadura, 10600 Plasencia, Cáceres, Spain; aperpic@unex.es (A.M.P.P.); pgvicky@unex.es (M.V.C.-M.); marinaf@unex.es (M.F.-J.); 2 Department of Nursing, Physiotherapy and Occupational Therapy, Faculty of Health Sciences, University of Castilla la Mancha, 45600 Talavera de la Reina, Toledo, Spain; julia.villar@uclm.es; 3 Escola Superior de Saúde Dr Lopes Días, Polytechnic Institute of Castelo Branco, 6000-084 Castelo Branco, Portugal; jbelo@ipcb.pt; 4 Department of Anatomy, Cellular Biology and Zoology, University Centre of Plasencia, University of Extremadura, 10600 Plasencia, Cáceres, Spain

**Keywords:** podiatric disorders, sports activity, prevalence

## Abstract

**Background/Objectives**: This study explores the prevalence of podiatric disorders in relation to factors such as gender, age, and the type of sports activity. Understanding these elements is crucial for implementing effective prevention strategies. **Methods**: The sample consisted of 70 participants, aged 12 to 30, with 71.4% youths and 28.6% adults, including 50 Portuguese and 20 Spanish individuals, who practiced either terrestrial or aquatic sports at varying training intensities. Statistical analyses were performed on data collected from athletes using chi-square tests and proportion tests. Variables such as exercise intensity and sport type (terrestrial vs. aquatic) were examined. **Results**: Skin disorders were more frequent in men (70.2%) compared to women (29.8%). Regarding age, 70% of individuals aged 19 to 30 years presented dermatoses, compared to 36% in the 12 to 18-year-old group. Exercise intensity also had an impact: 53.8% of athletes engaging in moderate activity exhibited keratoses, compared to 30.8% of those practicing intense activity. Additionally, athletes in terrestrial sports showed an average of 5.2 podiatric disorders, significantly higher than the 3.2 average in aquatic sports. Specifically, terrestrial athletes have a higher prevalence of pinch callus (84.6%) and hyperkeratosis on metatarsal heads (85.7%), while aquatic athletes have more onychomycosis (91.7%) and less hyperkeratosis. A higher prevalence of rotated toes (61.4%) and subungual hematoma (90.9%) was also observed in terrestrial athletes. **Conclusions**: The prevalence of podiatric disorders is significantly related to gender, age, and the type of physical activity. Men and young adults are more prone to dermatoses, while athletes engaging in moderate intensity activity and those athletes in terrestrial sport face a higher risk of podiatric issues. These findings highlight the need for prevention and treatment strategies in relation to the specific characteristics of each group.

## 1. Introduction

Physical exercise is widely recognized for its mental and physical health benefits, supported by scientific evidence [1,2]. However, all sports activities carry risks [3], including injuries, pathologies, or specific alterations that vary according to the type of sport [3,4,5,6,7], the intensity, frequency, duration of the exercise, and the technique used [8]. These risks increase if sports activities are practiced inappropriately or without supervision, and the environment in which they are performed can also influence the occurrence of injuries [7,9,10].

The 2022 Spanish Survey of Sports Habits revealed that 57.3% of participants over the age of 15 participated in sports activities during the last year, reflecting a 4% increase compared to 2015, indicating a shift toward a less sedentary lifestyle, as seen in other countries [11,12,13]. Nevertheless, globally, it is estimated that almost one-third of adults do not meet the recommended levels of physical activity, highlighting the need for effective strategies to combat physical inactivity and achieve the 2030 target [14]. This increase in sports participation could be associated with a rise in injuries, pathologies, and related alterations, reinforcing the importance of prevention. Foot injuries are particularly frequent [14] due to their fundamental role in weight distribution during terrestrial sports [5] and propulsion in aquatic sports [15]. The variability in foot function depending on the sport can lead to specific podiatric disorders, considering that pre-existing podiatric conditions can increase the risk of injuries during sports [15]. Sports like tennis and soccer show a higher incidence of lower limb injuries compared to the upper body [6,16]. Additionally, terrestrial and/or impact sports are more related to skin, nail, traumatic, deforming, or muscular foot pathologies [4,15], while aquatic sports are associated with a higher incidence of contagious conditions like mycosis [10].

Despite these risks, physical exercise, including aquatic sports, has shown significant benefits, such as reducing muscle pain, improving physical function, and enhancing the quality of life in adults with musculoskeletal conditions [17,18]. These benefits have led health authorities to promote physical exercise in all forms, as its advantages outweigh the associated risks, and many podiatric injuries can be prevented with proper safety measures [12]. Although the benefits are clear, it is essential to understand the specific pathologies of each sport and their impact on the foot to implement preventive measures that reduce their incidence [8,19]. This study will also examine variables such as gender, sports intensity, and age ranges to determine which activities present the most associated alterations and which are specific to each sport, enabling more effective prevention strategies. Direct comparisons of pathologies in terrestrial and aquatic sports activities are uncommon [17,20]. Many studies focus on injuries specific to each sport or the surface where the activity is performed without considering both groups simultaneously [6,21]. Although injuries and pathologies in both terrestrial and aquatic sports have been investigated separately [5,22], and the impact of different surfaces has been studied [23], there is a lack of comprehensive studies specifically addressing foot pathology. Therefore, this study aims to analyze podiatric conditions in participants of terrestrial and aquatic sports, identifying statistically significant differences in the prevalence of these conditions. It will also examine variables such as gender, sports intensity, and age ranges to determine which activities present more alterations and which are specific to each sport, allowing for more effective prevention strategies.

## 2. Materials and Methods

### 2.1. Study Type, Permissions, and Inclusion Criteria

A cross-sectional descriptive multicenter study was designed, it was and conducted at the University Center of Plasencia (University of Extremadura, Spain) and the Escola Superior de Saúde Dr. Lopes Dias (Polytechnic Institute of Castelo Branco, Portugal), from September 2022 to August 2023.

This study was authorized by the Bioethics Committee of UEx (Registry No.: 21/2022) and the direction was guided the Portuguese center, which accepted the UEx bioethics committee’s approval. This study adhered to the ethical principles of the Declaration of Helsinki and the current biomedical research legislation in both countries (Law 14/2007, of 3 June). All participants were informed, voluntarily joined the study, and signed to show their informed consent. Minors’ consent forms were signed by their parents or legal guardians. Participants were required to be between 12 and 30 years old, provide the necessary information, and sign the informed consent form. To be classified as athletes, participants had to engage exclusively in a terrestrial or aquatic sport, training more than three hours weekly with a minimum frequency of twice per week for at least the past year [24]. Terrestrial sports included tennis, soccer, and running, while swimming was the aquatic sport. All participants were required to use proper footwear for their sport (terrestrial) and not have received or be receiving podiatric or medical treatment or have chronic foot conditions that could compromise podiatric health assessments.

### 2.2. Methodology

Following the protocol established by Pérez Pico et al. [15], an experienced examiner conducted all evaluations and collected sociodemographic and clinical data from the participants. This included personal, podiatric history, medication use, allergies, and any relevant treatments to provide context for the participants’ podiatric disorders. The podiatric evaluation was conducted through direct observation, identifying and recording skin, nail, and deformity alterations. To detect fungal infections, Wood’s lamp and mycological cultures were used to confirm the presence of fungi [25]. Collected data were organized, cleaned, and statistically analyzed.

### 2.3. Sample and Variables Analyzed

The sample consisted of 70 participants, divided into two age groups: youths aged 12 to 18 (71.4%) and adults aged 19 to 30 (28.6%), with a mean age of 17.5 ± 4.8 years. Regarding nationality, 20 participants were Spanish (15.6 ± 2.2 years; 28.6%) and 50 Portuguese (18.4 ± 5.3 years; 71.4%). In terms of gender, 29 were women (15.2 ± 3.7 years; 41.4%) and 41 were men (19.2 ± 4.8 years; 58.6%). Regarding sports activity, 34 practiced terrestrial sports (19.7 ± 5 years; 48.6%), and 36 practiced aquatic sports (15.5 ± 3.5 years; 51.4%). None were professional athletes. In terms of training intensity, 31 participants trained at high intensity (16.3 ± 4.4 years), 20 at moderate intensity (20.7 ± 4.8 years), and 19 at low intensity (16.3 ± 4 years). Athletes in terrestrial sport trained an average of 6.2 ± 4.7 h per week (7.7 ± 4.8 h for men and 2.7 ± 1.9 h for women), while aquatic athletes trained an average of 9.9 ± 4.3 h per week (9.6 ± 3.1 h for men and 9.9 ± 4.4 h for women).

Both qualitative and quantitative variables were analyzed. The qualitative variables included country of origin, age groups, gender, and the presence of podiatric disorders (skin, nail, and deformities). The intensity of physical activity was categorized into three levels, low, moderate, and high intensity, based on the time spent exercising per week. Low intensity for athletes who engaged in physical activity for 3 h or less per week, moderate intensity for those who exercised for more than 3 h but less than 10 h per week, and high intensity for athletes who engaged in physical activity for more than 10 h per week. Disorders were grouped into the following four categories: (1) dermatoses (any skin disorder that does not involve hyperkeratosis); (2) keratoses (any skin disorder primarily involving hyperkeratosis); (3) onychopathies (any nail disorder); and (4) toes deformities (any deformity in the toes). Additionally, the presence of infections in the skin and/or nails was assessed through laboratory analysis. All variables were categorized as either the presence (yes) or absence (no) of a disorder. Initially, the presence or absence of disorders were recorded in the groups, meaning if one disorder was found it was categorized as “yes”, and if no disorders were found it was categorized as “no”. Subsequently, each specific disorder observed on the participants’ feet was documented individually within each group (Appendix A.1).

The quantitative variables included age (in years), the total number of disorders in each group of podiatric disorders, and the total number of disorders present on the feet. Age was grouped into two categories (youths and adults) and made a dichotomous variable (Appendix A.1).

### 2.4. Statistical Data Analysis

Statistical analysis was conducted using IBM-SPSS Statistics for Windows (version 29.0.1.0). Qualitative variables were analyzed using the chi-square test for independence, with a 5% significance level, and the Z-test for comparing proportions.

For quantitative variables, data were cleaned by removing outliers, normality was checked, and means were analyzed. Student’s *t*-test or the Mann–Whitney U test were applied, maintaining a 5% significance level in each case. Additionally, effect size was calculated using Cohen’s d for parametric tests and the effect size R for non-parametric tests to reflect the strength of association between variables or the effect size [25].

## 3. Results

### 3.1. Risk Factors Associated with Podiatric Disorders

#### 3.1.1. Gender and Prevalence of Podiatric Disorders

Analyzing the prevalence of podiatric disorders by gender revealed a higher incidence of skin disorders in men (70.2%) compared to women (29.8%). Specifically, an association was found between skin disorders and gender, with 80.5% of men presenting a skin disorder compared to 48.3% of women (*p*-value = 0.009). The difference in proportions was −32.2%, which was statistically significant (*p*-value = 0.002), confirming that skin disorders are significantly more common in men than in women, highlighting the importance of considering gender in the analysis of these disorders.

Regarding dermatoses, a higher prevalence was also found in men (75% of cases) compared to women (25%). Specifically, 58.5% of men had a dermatopathy compared to 27.6% of women (*p*-value = 0.015). The difference in proportions was −22.2%, statistically significant (*p*-value = 0.029), further supporting that dermatoses are more common in men than in women. Desquamation was more common in men (75%) than in women (25%), with a significant difference (*p*-value = 0.044). Additionally, erythrasma was only observed in men. Hyperkeratosis on the dorsal surface of the toes was also more frequent in men (88.9%) than in women (11.1%) (*p*-value = 0.020). Finally, signs of onychomycosis were more common in men (75%) than in women (25%) (*p*-value = 0.013).

#### 3.1.2. Age Groups and Prevalence of Podiatric Conditions

Analyzing the relationship between age groups and the prevalence of podiatric conditions revealed the following associations:

- General dermatoses: 36% of younger participants had dermatopathy compared to 70% of adults (*p*-value = 0.016). While the younger group had more total cases, the prevalence was significantly higher in adults, suggesting a greater tendency toward dermatoses in older individuals. The proportion difference between age groups was −34%, statistically significant (*p*-value = 0.005), confirming that dermatoses are more common in the older group.
- General keratoses: Both age groups were equally affected by keratoses (50% in both). However, within the groups, 26% of younger participants had keratopathy compared to 65% of adults (*p*-value = 0.005). Although the younger group has more participants overall, the prevalence of keratoses is significantly higher in the adult group, indicating a greater tendency in older individuals. The proportion difference was −39%, statistically significant (*p*-value = 0.001), reinforcing that keratoses are more prevalent in the older group.
- Skin disorders: 59.6% of younger participants had skin alterations, compared to 40.4% of adults. Specifically, based on age ranges, 95% of adults had skin disorders, compared to 56% of younger participants (*p*-value = 0.002). However, the prevalence within the adult group is much higher, indicating a greater tendency toward skin disorders in this group, despite the younger group having a higher total number of cases. The proportion difference was −39%, statistically significant (*p*-value < 0.001), confirming that skin disorders are more prevalent in older individuals. A significant increase in the prevalence of tailor’s bunion was observed in the 19 to 30 age group (14.3%) compared to the 12 to 18 age group (1.7%) (*p*-value = 0.020). Erythrasma was only detected in the 19 to 30 age group (17.9%). A higher prevalence of subungual hematomas was also found in the 19 to 30 age group (35.7%) compared to the 12 to 18 age group (6.9%) (*p*-value = 0.001).

#### 3.1.3. Sports Intensity and Prevalence of Podiatric Disorders

Analyzing the relationship between sports intensity and podiatric disorders revealed the following associations:

- General skin disorders: Athletes training at high intensity showed the highest percentage of skin disorders (40.4%), followed by moderate intensity (38.3%) and low intensity (21.3%). Specifically, 90% of moderate-intensity athletes had skin disorders, compared to 61.3% of high-intensity and 52.6% of low-intensity athletes (*p*-value = 0.030). The proportion difference between groups showed that: between low and moderate intensity, the difference was −37.4% (not statistically significant); between low and high intensity, the difference was −8.7% (not statistically significant); and between moderate and high intensity, the difference was 28.7%, and these were statistically significant (*p*-values = 0.005, 0.274, and 0.012, respectively). These results suggest a relationship between training intensity and the prevalence of skin disorders, although it is worth noting that insufficient evidence was found to confirm this relationship among low- and high-intensity participants.
- Keratoses: Moderate-intensity athletes had the highest percentage of keratoses (53.8%), followed by high intensity (30.8%) and low intensity (15.4%). Specifically, 70% of moderate-intensity athletes had keratoses, compared to 25.8% of high-intensity and 21.1% of low-intensity athletes (*p*-value = 0.001). The proportion difference between groups showed that between low and moderate intensity, the difference was −48.9%, and between moderate and high intensity, the difference was 44.2%, and both were statistically significant (*p*-value = 0.001; 0.002). These results confirm the relationship between training intensity and the prevalence of keratoses.

Similarly, in the case of erythrasma, it was predominantly observed in moderate-intensity athletes (80%) compared to low-intensity athletes (20%), with a *p*-value of 0.024. Mycosis showed a trend toward statistical significance in high-intensity athletes (*p*-value = 0.058). On the other hand, metatarsal heads showed a higher prevalence in moderate-intensity athletes (57.1%) (*p*-value = 0.030). Regarding hyperkeratosis on the dorsal surface of the toes, this condition was more common in moderate-intensity athletes (77.8%), with a *p*-value of 0.002. Finally, signs of onychomycosis were more frequent in athletes with a higher training intensity, with a *p*-value of 0.029. It is important to note that due to the presence of empty cells, these results should be interpreted with caution.

### 3.2. Differences in the Number of Podiatric Disorders Based on Sports Activity

The average number of podiatric disorders observed across all athletes was 4.2 ± 2.9 (See Table 1). When analyzing based on the type of sport, the average number of podiatric disorders was higher in athletes in terrestrial sports (4.7 ± 2.6) compared to aquatic sports (3.2 ± 2) (*p*-value = 0.007), indicating a statistically significant difference between the two groups. Moreover, the effect size was large (Cohen’s d = 2.8), suggesting the sample is robust enough to support these conclusions.

Regarding toe alterations, the average number for all participants was 1.9 ± 1.2 (see Table 1). For athletes in terrestrial sports, the average was 2.1 ± 1.3, and for aquatic sports, it was 1.5 ± 1.1, although this difference was not significant (*p*-value = 0.092).

As for skin disorders (Q + D), the average number for all participants was 1.4 ± 1.6 (See Table 1). In terrestrial sports, the average was 2 ± 1.9, while in aquatic sports it was 0.9 ± 1, with a statistically significant difference (*p*-value = 0.012). However, the effect size (r = −0.3) suggests a moderate effect, indicating that the sample might not be large enough for high confidence in the results and conclusions.

When analyzing individual alterations, the average number of keratoses for all participants was 0.6 ± 0.9 (see Table 1). In athletes participating in terrestrial sports, the average was 1.1 ± 1.1, while in aquatic sports it was 0.2 ± 0.4, with a statistically significant difference (*p*-value < 0.001). The effect size (r = −0.5) indicates a moderate effect, suggesting that the sample might not be large enough for high confidence in the results and conclusions. As for dermatoses, the overall average was 0.8 ± 1.1 (see Table 1). In athletes in terrestrial sports, the average was 1 ± 1.2, and in aquatic sports, it was 0.7 ± 0.9, with no significant difference (*p*-value = 0.271). Lastly, the average number of nail disorders for all participants was 1 ± 1.1 (see Table 1). In athletes in terrestrial sports, it was 1.1 ± 1.3, and in aquatic sports, 0.9 ± 0.8, with no statistically significant differences (*p*-value = 0.742).

### 3.3. Comparison Between Types of Sports and the Prevalence of Podiatric Disorders

When analyzing the prevalence of podiatric disorders in athletes in terrestrial sports and athletes in aquatic sports, a dependent relationship was found in several variables (see Table 2). Athletes in terrestrial sports had a higher percentage of general toe alterations (55.9% vs. 44.1%) (*p*-value = 0.007) (see Table 2), rotated toes (61.4% vs. 38.6%) (*p*-value = 0.007), keratoses (76.9% vs. 23.1%) (*p*-value < 0.001), pinch callus (84.6% vs. 15.4%) (*p*-value = 0.005), hyperkeratosis on the heads of metatarsals (85.7% vs. 14.3%) (*p*-value = 0.002), nail striations (81.8% vs. 18.2%) (*p*-value = 0.022), and subungual hematomas (90.9% vs. 9.1%) (*p*-value = 0.003). In contrast, onychomycosis was more prevalent in aquatic athletes (91.7% vs. 8.3%) (*p*-value = 0.003). The differences in proportions between athletes in terrestrial sports and athletes in aquatics sport were 42.2% in toe deformities, 24.8% in keratoses, 26.8% in pinch callus, 29.7% in hyperkeratosis on metatarsal heads, 20.9% in nail striations, and 27.6% in onychomycosis; all statistically different (*p*-values < 0.001; 0.002; 0.002; <0.001; 0.008; and 0.001) (see Table 2).

In terms of the presence of skin mycosis, the chi-square test did not reveal a significant association between athlete groups (*p*-value = 0.068) (see Table 2). However, the proportion contrast showed a significant difference (*p*-value = 0.036), suggesting specific differences between the groups that the overall test did not detect. This difference highlights the need for deeper analysis, as specific patterns within the study groups may exist. No significant differences were observed in the other podiatric disorders in relation to the type of sport practiced (Appendix A.2).

### 3.4. Comparison Between Sex, the Prevalence of Podiatric Disorders, and the Sports Activity Performed

When analyzing the prevalence of podiatric disorders based on sex in each sports activity, a significant relationship was found in some variables, such as the presence of infraductus toes, with women showing a higher prevalence (60% vs. 40%) and this difference being significant in the group of athletes participating in terrestrial sports (*p*-value = 0.034) and with a statistically significant difference in proportions (*p*-value = 0.006). Regarding general skin disorders, men were more affected in the terrestrial sports group (84.6% vs. 15.4%), with this difference being statistically significant (*p*-value = 0.003) and with a statistically significant difference in proportions (*p*-value ≤ 0.001).

### 3.5. Comparison Between Age Ranges, Podiatric Disorders, and Type of Sports Activity

When comparing the prevalence of podiatric disorders according to age ranges and the type of sports activity, no clear relationship could be established for any of the studied alterations. Although the chi-square test indicated significance in several alterations, the expected counts being less than five suggest that a larger sample size is needed to obtain more reliable results.

### 3.6. Sports Intensity, Podiatric Disorders, and Type of Sports Activity

Similarly, when comparing podiatric disorders based on the intensity of the sports activity and the type of sport, no clear relationship was observed with any of the studied alterations. The expected counts being less than five also indicate the need for a larger sample size to achieve more conclusive results.

## 4. Discussion

The foot, as a fundamental part of the human structure, plays a crucial role in locomotion and balance. However, its functions vary considerably depending on the type of physical activity performed. In daily life, the demands on the foot are minimal, which contrasts with the specific demands of sports, both terrestrial and aquatic. In terrestrial sports, the foot bears the body’s weight, absorbs impacts, provides stability, and facilitates dynamic movement [26]. In contrast, in aquatic sports, such as swimming, the foot does not face similar loads but adapts to optimize propulsion and floatability [27,28].

This variability in foot functions suggests that podiatric disorders may differ depending on the habitual physical activity. In a previous study conducted by this group of researchers, foot injuries were observed during a 30 km hike. The participants presented various foot alterations, such as blisters, grazes, chafing, crevices, and calluses, reflecting the consequences of practicing terrestrial sports [15]. In our research, we observed that athletes present a diversity of alterations, including dermatoses, keratoses, and toe deformities. The average number of podiatric disorders was 4.2 ± 2.9, higher in athletes partaking in terrestrial sports. This indicates that terrestrial sports carry a higher risk of injury due to biomechanical demands, although the moderate effect size suggests the need to expand the sample for greater reliability in the results [29].

The analysis of factors such as sex, age, and sports intensity revealed that men exhibit more dermal alterations, probably due to differences in footwear type and training conditions [30]. Likewise, skin disorders were associated with older athletes, which could reflect physiological changes or the cumulative impact of daily footwear [31].

Regarding the intensity of physical activity, it was found that both low and moderate intensity are related to skin disorders, while keratoses showed a stronger correlation with moderate and high intensities. These findings highlight the need to consider training intensity and other external factors, such as footwear type and athletes’ physiological adaptation, when evaluating podiatric health [32].

Furthermore, specific alterations were identified based on the type of sport practiced by the participants. Generally, terrestrial sports are associated with deformities such as rotated toes, hyperkeratosis, and subungual hematomas, while aquatic sports show a higher prevalence and incidence of onychomycosis and dermal infections [33]. These results, aligning with previous studies, suggest that the specific environmental and biomechanical conditions of each sport influence the type of injuries [29,34].

Foot health is a priority for athletes, who often seek to improve their performance [34,35,36,37]. Therefore, it is essential to develop prevention strategies tailored to the particularities of each group of athletes, considering their activity intensity level and the specific alterations they face.

We recommend the implementation of periodic individualized check-ups to monitor and treat podiatric disorders in both groups of athletes, terrestrial and aquatic, recognizing that each group faces a higher risk of certain types of conditions. The intervention of a healthcare professional is crucial, as their expertise enables appropriate diagnosis and treatment, which could prevent long-term injuries [15,38].

## 5. Conclusions

Podiatric disorders are common in the athletes studied, with an average of 4.2 total alterations. These include skin problems, dermatoses, keratoses, and nail and toe deformities.

Risk factors such as sex, age, and training intensity are significantly related to the appearance of these alterations. In particular, being male, older, and participating in more intense training increases the probability of developing podiatric problems. It is essential to consider these factors in future research on podiatric health in athletes.

Athletes in terrestrial sport have a higher frequency of alterations, such as rotated toes, hyperkeratosis in central metatarsals, pinch calluses, longitudinal striations on nails, and subungual hematomas, while onychomycosis is more prevalent among aquatic athletes. Additionally, specific trends in alterations were identified based on sex, such as infraductus toes in women and general skin disorders in men in the terrestrial sports group.

To promote podiatric health and prevent future injuries, we recommend implementing prevention programs from an early age, as well as personalized periodic check-ups for athletes. This will allow for proactive management of alterations and optimize sports performance.

## Figures and Tables

**Table 1:** Analysis of podiatric disorders in athletes in terrestrial sport and athletes in aquatic sport with comparison tests (Student’s *t*-test or Mann–Whitney) and effect size.

General Quantitative Variables	All Athletes	Terrestrial Sports	Aquatic Sports	*p*-Value	Effect Size
Total number of podiatric alterations	4.2 ± 2.9	4.7 ± 2.6	3.2 ± 2	0.007 *	2.8 †
Number of toe alterations	1.9 ± 1.2	2.1 ± 1.3	1.5 ± 1.1	0.092	n.a.
Number of skin disorders (Q + D)	1.4 ± 1.6	2 ± 1.9	0.9 ± 1	0.012 **	−0.3 ‡
Number of keratoses	0.6 ± 0.9	1.1 ± 1.1	0.2 ± 0.4	<0.001 **	−0.5 ‡
Number of dermatoses	0.8 ± 1.1	1 ± 1.2	0.7 ± 0.9	0.271	n.a.
Number of nail disorders	1 ± 1.1	1.1 ± 1.3	0.9 ± 0.8	0.742	n.a.

n.a. = not available, * = statistically significant with Student’s *t*-test, ** = statistically significant with Mann–Whitney test, Q + D = combined variable for keratoses and dermatoses. † = effect size using d of Cohen, ‡ = effect size using r.

**Table 2:** Relationship between podiatric disorders and the type of physical activity performed. Comparison with chi-square test and proportion contrast.

	Terrestrial Sports	Aquatic Sports	*p*-Value (χ^2^)	*p*-Value
Total Keratoses (n = 26)	76.9% (n = 20)	23.1% (n = 6)	<0.001 *	0.002 *
Total toe alterations (n = 59)	55.9% (n = 33)	44.1% (n = 26)	0.007 *	<0.001
Pinch callus (n = 13)	84.6% (n = 11)	15.4% (n = 2)	0.005 *	0.002 *
Hyperkeratosis on metatarsal heads (n = 14)	85.7% (n = 12)	14.3% (n = 2)	0.002 *	<0.001
Subungual hematoma (n = 11)	90.9% (n = 10)	9.1% (n = 1)	0.003 *	0.001 *
Onychomycosis in lab tests (n = 12)	8.3% (n = 1)	91.7% (n = 11)	0.003 *	0.001 *
Lab-tested mycosis (n = 12)	25% (n = 3)	75% (n = 9)	0.068	0.036 *
Rotated toes (n = 44)	61.4% (n = 27)	38.6% (n = 17)	0.007 *	0.003 *
Infraductus toe (n = 19)	52.6% (n = 10)	47.4% (n = 9)	0.790	0.339

n = sample size, χ^2^ = chi-square, % = percentage, * = statistically significant.

## Data Availability

Data are contained within the article.

## Appendix A

### Appendix A.1

**Table A1:** Studied qualitative and quantitative variables.

Qualitative Variables	Category
Study Group	Athletes in terrestrial sports/Athletes in aquatic sports
Country	Spain/Portugal
Sex	Male/Female
Age Range	Youth/Adult
Intensity of Sports Activity	High (>10 h)/Moderate (>3 > 10 h)/Low (>10 h)
Group Variables	Category
Skin Disorders (Keratoses and Dermatoses)	Presence of any dermal alteration (Yes) Absence (No)
Keratoses	Presence of any keratinization alteration (Yes) Absence (No)
Dermatoses	Presence of any dermal alteration without keratinization alteration (Yes) Absence (No)
Onychopathies	Presence of any nail alteration (Yes) Absence (No)
Toes Deformities	Presence of any digital alteration (Yes) Absence (No)
Keratoses	Category
Pinch callus	Presence (Yes) Absence (No)
Hyperkeratosis in Metatarsal Heads	Presence (Yes) Absence (No)
Hyperkeratosis in Heel	Presence (Yes) Absence (No)
Hyperkeratosis in Dorsal Toes	Presence (Yes) Absence (No)
Dermatoses	Category
Papilloma	Presence (Yes) Absence (No)
Hyperhidrosis	Presence (Yes) Absence (No)
Edema	Presence (Yes) Absence (No)
Xerosis	Presence (Yes) Absence (No)
Desquamation	Presence (Yes) Absence (No)
Erythrasma	Presence (Yes) Absence (No)
Infectious Skin Pathology	Category
Mycosis	Presence (Yes) Absence (No)
Onychopathies	Category
Onycholysis	Presence (Yes) Absence (No)
Ingrown Nail	Presence (Yes) Absence (No)
Striations (General)	Presence (Yes) Absence (No)
Beau’s Lines or Transverse Lines	Presence (Yes) Absence (No)
Longitudinal Striations	Presence (Yes) Absence (No)
Leukonychia	Presence (Yes) Absence (No)
Xanthonychia	Presence (Yes) Absence (No)
Convoluted Nail	Presence (Yes) Absence (No)
Cracked Nail	Presence (Yes) Absence (No)
Subungual Hematoma	Presence (Yes) Absence (No)
Total Onychodystrophy	Presence (Yes) Absence (No)
Infectious Nail Pathology	Category
Onychomycosis	Presence (Yes) Absence (No)
Toes Deformities	Category
Tailor’s Bunion	Presence (Yes) Absence (No)
Rotated Toes	Presence (Yes) Absence (No)
Supraductus Toe	Presence (Yes) Absence (No)
Infraductus Toe	Presence (Yes) Absence (No)
Claw or Hammer Toe	Presence (Yes) Absence (No)
Syndactyly	Presence (Yes) Absence (No)
Hallux Valgus	Presence (Yes) Absence (No)
Quantitative Variables	Category
Age	Years
Training Hours	Weekly Training Hours
Total Number of Foot Alterations	Sum of podiatric disorders present in the participant’s foot
Number of Dermatoses Present	Sum of Dermatoses present in the participant’s foot
Number of Keratoses Present	Sum of Keratoses present in the participant’s foot
Number of Onychopathies Present	Sum of Onychopathies present in the participant’s foot
Number of Toes Deformities Present	Sum of Toes Deformities present in the participant’s foot

### Appendix A.2

**Table A2:** Prevalence of podiatric disorders in participants based on physical activity. * = statistically significant with Student’s *t*-test, ** = statistically significant with Mann–Whitney test.

Presence of Podiatric Disorders	TS (n)	AS (n)	*p*-Value χ^2^	Proportions Test
Skin Disorders (Keratoses and Dermatoses) (n = 47)	55.3% (26)	44.7% (21)	0.131	0.053
Keratoses (n = 26)	76.9% (20)	23.1% (6)	<0.001 *	0.002 *
Dermatoses (n = 32)	53.1% (17)	46.9% (15)	0.632	0.242
Onychopathies (n = 43)	48.8% (21)	51.2% (22)	1.000	0.478
Toes Deformities (n = 59)	55.9% (33)	44.1% (26)	0.007 *	<0.001
Keratoses				
Pinch callus (n = 13)	84.6% (11)	15.4% (2)	0.005 *	0.002 *
Hyperkeratosis in Metatarsal Heads (n = 14)	85.7% (12)	14.3% (2)	0.002 *	<0.001
Hyperkeratosis in Heel (n = 7)	71.4% (5)	28.6% (2)	0.253 **	0.101
Hyperkeratosis in Dorsal Toes (n = 9)	88.9% (8)	11.1% (1)	0.012 **	0.005 **
Dermatoses				
Skin Color Change (n = 4)	75% (3)	25% (1)	0.350 **	0.138 **
Papilloma (n = 2)	50% (1)	50% (1)	1.000 **	0.484 **
Hyperhidrosis (n = 9)	66.7% (6)	33.3% (3)	0.300 **	0.122 **
Xerosis (n = 5)	80% (4)	20% (1)	0.192 **	0.072 **
Desquamation (n = 15)	60% (9)	40% (6)	0.389	0.159
Infectious Skin Pathology				
Erythrasma (n = 5)	100% (5)	0% (0)	0.023 **	0.008 **
Mycosis Lab Test (n = 12)	25% (3)	75% (9)	0.112	0.036 *
Onychopathies				
Onycholysis	50% (5)	50% (5)	1.000	0.461
Striations (general)	81.8% (9)	18.2% (2)	0.022	0.008 *
Beau’s lines	71.4% (5)	28.6% (2)	0.253	0.101
Longitudinal striations	100% (5)	0% (0)	0.023	0.008 *
Leukonychia	12.5% (1)	87.5% (7)	0.056	0.015 *
Xanthonychia	0% (0)	100% (2)	0.493	0.082
Ingrown nail	57.1% (4)	42.9% (3)	0.706	0.316
Cracked nail	0% (0)	100% (1)	1.000	0.164
Subungual hematoma	90.9% (10)	9.1% (1)	0.003	0.001 *
Infectious Pathology in Nails				
Onychomycosis (laboratory)	8.3% (1)	91.7% (11)	0.003	0.001 *
Toes Deformities				
Tailor’s bunion	100% (5)	0% (0)	0.023	0.008 *
Rotated toes	61.4% (27)	38.6% (17)	0.007	0.003 *
Supraductus toe	50% (5)	50% (5)	1.000	0.461
Infraductus toe	52.6% (10)	47.4% (9)	0.790	0.339
Claw or hammer toe	45.5% (10)	54.5% (12)	0.800	0.362
Syndactyly	50% (1)	50% (1)	1.000	0.484
Hallux valgus	57.1% (12)	42.9% (9)	0.437	0.174
